# Mycorrhizal fungus BJ1, a new species of *Tulasnella* sp.: its biological characteristics and promoting effect on seed germination of *Bletilla striata*

**DOI:** 10.3389/fpls.2025.1542585

**Published:** 2025-02-21

**Authors:** Zhengmei Li, Yueyu Ye, Xia Wang, Suyu Peng, Beibei Chen, Shiqing Li, Haimin Chen, Dongfeng Yang, Fusheng Jiang, Chunchun Zhang, Meiya Li

**Affiliations:** ^1^ College of Pharmaceutical Sciences, Zhejiang Chinese Medical University, Hangzhou, Zhejiang, China; ^2^ College of Life Sciences, Zhejiang Chinese Medical University, Hangzhou, Zhejiang, China; ^3^ Key Laboratory of Plant Secondary Metabolism and Regulation of Zhejiang Province, College of Life Sciences and Medicine, Zhejiang Sci-Tech University, Hangzhou, Zhejiang, China; ^4^ Academy of Chinese Medical Sciences, Zhejiang Chinese Medical University, Hangzhou, Zhejiang, China

**Keywords:** symbiotic germination, mycorrhizal fungus, *Bletilla striata*, *Tulasnella* sp., indoleacetic acid

## Abstract

Mycorrhizal fungi have been shown to promote seed germination and seedling growth in Orchidaceae plants. In the present study, a mycorrhizal fungus designated as BJ1 was isolated from the roots of *Bletilla striata* (Thunb.) Reiehb.f. Fluorescence staining and morphological analysis revealed that this fungus exhibited characteristics highly similar to those of *Tulasnella*. Subsequently, the strain was confirmed as a new strain of *Tulasnella* through sequencing and phylogenetic analysis of four loci: the internal transcribed spacer region ITS1-ITS4 (ITS), ATP synthase (C14436), glutamate synthase (C4102), and ATP deconjugase (C3304). Additionally, we investigated the *in vitro* biological activity of strain BJ1 and its effects on germination and growth of *B. striata* seeds. The results indicated that BJ1 is capable of producing plant cell-degrading enzymes, including pectinase and protease. Furthermore, it demonstrates an ability to solubilize inorganic phosphorus and synthesize indoleacetic acid (IAA). Nevertheless, it does not exhibit laccase activity or possess the capacity to produce siderophores, nor can it solubilize organic phosphorus. Microscopic observations revealed that strain BJ1 mainly colonizes the base of the *B. striata* protocorm, thereby enhancing seed germination, growth, and expansion. Notably, by the fourth week of germination, 74.23% of seeds in the symbiotic group had developed to stage 5, a significantly higher proportion compared to 50.43% in the non-symbiotic group. Additionally, the length, width, and fresh weight of seeds in the symbiotic group were 2.2 times, 1.8 times, and 3.7 times greater than those in the non-symbiotic group, respectively. Furthermore, by adding L-tryptophan as a substrate during co-cultivation with BJ1, there was a significant enhancement in IAA synthesis capability; this also led to a marked acceleration in the symbiotic germination process of *B. striata* seeds. These results suggest that strain BJ1 holds significant potential for application in the artificial propagation of *B. striata* seedlings. It can enhance propagation efficiency and improve seedling quality, thereby playing a crucial role in the conservation and sustainable development of germplasm resources of endangered orchids.

## Introduction

*Bletilla striata* (Thunb.) Reiehb.f. (*B. striata*) is a perennial herb belonging to the Orchidaceae family, mainly distributed in Japan, South Korea and coastal areas of China. As a traditional Chinese herbal medicine, the underground part (tuber) of *B. striata* has been utilized for thousands of years due to its properties such as astringency, hemostasis, detumescence, and promotion of granulation. This herbal remedy is predominantly employed in the treatment of traumatic hemorrhage, hematemesis, chapped skin, swelling, and ulcer (Kong et al., 2021; Zhao, 1963). *B. striata* not only possesses significant medicinal value but also serves as an essential raw material in industrial production (Pan et al., 2018).

Previously, *B. striata* available on the market was mainly sourced from wild collections. This situation can be attributed to the low rate of natural seedling formation due to inadequate seed development, and the underdeveloped artificial propagation techniques. However, with the expansion of market demand, overexploitation of wild *B. striata* populations has led to a significant depletion of its wild resources. In recent years, advancements in artificial breeding technology have led to the establishment of various seedling cultivation methods for *B. striata*, such as ramet propagation (Zhang et al., 2019; Ren et al., 2016), tissue culture techniques (Zou et al., 2013), seed direct seeding, and symbiotic germination, which have effectively mitigated resource shortages in the market (Pan et al., 2018). Ramet propagation is a crucial early-stage method characterized by simple operation procedures with high seedling rates and short cultivation periods (Ren et al., 2016). However, this approach suffers from low propagation efficiency; prolonged vegetative propagation through ramets can result in virus accumulation along with variety degradation that adversely affects yield quality (Zhang et al., 2019). Tissue culture offers higher propagation efficiency but incurs substantial costs related to equipment and labor, as well as involving multiple complex procedures (Zhang et al., 2019). Direct seeding technology is simple; however it incurs high management costs while yielding relatively low seedling rates. In comparison to these three aforementioned reproductive methods, symbiotic germination presents distinct advantages. This technique not only enhances the germination rate as well as both seedling success rates and transplant survival rates for *B. striata* seeds but also saves costs, thereby better aligning with market demands and simultaneously protecting wild *B. striata* resources (Pan et al., 2018; Zhang et al., 2005; Xu et al., 2019).

The earliest technology for symbiotic germination can be traced back to 1899, when Bernard discovered that fungal infection is essential for the germination and growth of orchid seeds. Fungi provide the necessary nutrients required for their germination, leading to the breakthrough establishment of symbiotic germination technique. This method plays a crucial role in both research and conservation efforts concerning Orchidaceae plants (Xu and Xu, 2007; Gao et al., 2019; Fan et al., 2003). Mycorrhizal fungi include ectomycorrhiza and endophytic mycorrhiza. Endophytic mycorrhizal fungi are those whose mycelia can invade the root cells of higher plants while coexisting harmoniously with them, thereby promoting plant growth (Zhao et al., 2021). The mycelium of mycorrhizal fungi penetrates plant root cells to form specialized structures such as arbuscular (the main site for nutrient transfer between fungi and plants) and vesicles (which form in cortical cells and act as storage organs for lipids and other resources), facilitating efficient nutrient exchange. Mycorrhizal fungi can engage in bidirectional nutrient exchange with plants through their intricate mycelial networks in the soil. Plants supply the fungi with fixed carbon, such as sugars, while the fungi provide essential minerals, thereby enhancing the efficiency of nutrient uptake by plants (Harley and Smith, 1983). Furthermore, Mycorrhizal fungi not only promote seed germination and growth but also enhance the utilization rates of phosphorus and potassium, secrete plant growth-regulating hormones, thereby promoting overall plant development and significantly improving host adaptability to adverse environmental conditions (Zhao et al., 2021; Yang et al., 2024; Miura et al., 2018). Furthermore, in certain medicinal plants, mycorrhizal fungi can also increase the content of their active components (Wang et al., 2023).

Orchidaceae seeds lack complete endosperm, making them challenging to germinate and grow under natural conditions. These seeds interact with microorganisms to form a unique symbiotic system for germination and growth (Yang et al., 2020). At present, a large number of symbiotic fungi have been isolated and identified from Orchidaceae plants. For example, Gao et al. (2019) identified *Tulasnella* sp.–a fungus capable of promoting seed germination in approximately forty different Orchidaceae species-while *Ceratobasidium* sp. has been shown to facilitate seed germination in over twenty species within this family. The symbiotic relationship between mycorrhizal fungi and Orchidaceae seeds plays an irreplaceable role in supporting their growth under natural conditions. As an effective strategy for conserving Orchidaceae resources, such fungi have been widely utilized in the reproduction and cultivation of these Orchidaceae plants (Batty et al., 2006).

*B. striata* exhibits seed germination challenges similar to those faced by other members of the Orchidaceae family. Therefore, finding mycorrhizal fungi that can facilitate the symbiotic germination and growth of *B. striata* seeds is essential for achieving efficient and rapid propagation of this species. Numerous studies have reported on mycorrhizal fungi that promote the germination and growth of *B. striata* seeds. For example, Guo and Xu (1992) found that four fungal species, including *Myeena osmundieola* Lange, could promote both seed germination and seedling development in *B. striata* seeds. Xu et al. (2019) investigated four different mycorrhizal fungi species regarding their effects on seed germination and seedling growth in *B. striata*; they found significant variations in symbiotic performance among different mycorrhizal fungi when interacting with *B. striata*.

In our current study, we isolated a mycorrhizal fungus designated as BJ1 from the roots of *B. striata*; it was identified as belonging to *Tulasnella* sp., exhibiting strong capabilities for promoting germination and growth. We systematically examined its biological characteristics-including enzyme activity related to parasitism and nutrient absorption-as well as its capacity for plant hormone production-and analyzed its impact on the symbiotic germination process of *B. striata* seeds over time. Our findings provide valuable technical support for the efficient cultivation of high-quality seedlings of *B. striata* in the future horticultural practices.

## Materials and methods

### Morphological and molecular identification of fungi

#### Morphological observation

**Figure 1:**
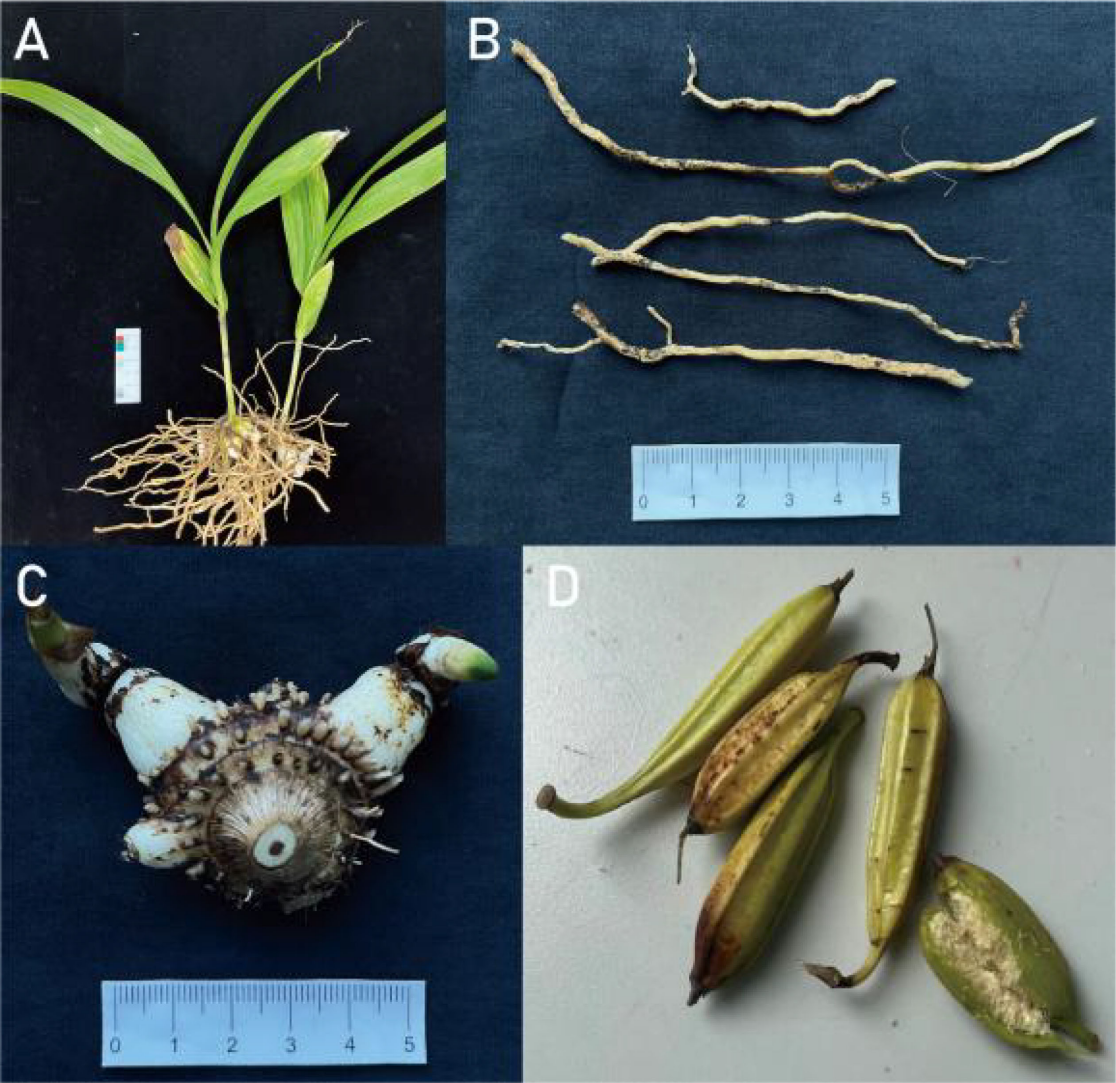
Biomorphology of *B striata*. *B striata* plant **(A)**, fibrous root **(B)**, tuber **(C)** and fruit clips **(D)**.

The strain BJ1 was isolated from the fibrous roots of *B. striata* (**Figure 1**; **Supplementary Figure S1**) and has been deposited at the China General Microbiological Culture Collection Center, with a preservation No. 40773. The BJ1 culture was transferred to potato dextrose agar (PDA) and incubated at 28°C for 1 week (Zhu et al., 2012; Daniel et al., 2022). The basic morphological characteristics of BJ1 colonies were observed and recorded, including color, morphology, growth rate, and cell characteristics. Then, the microstructure of hyphae was observed using both a light microscope and an electron microscope (field emission scanning electron microscopy, FESEM; SU8010, Japan), focusing on hyphal structure and cell morphology.

#### Fluorescence staining of fungal nuclei

The BJ1 strain was inoculated onto four different culture media: ½ FIM medium, E-medium, 3MN/Magali’s Modified Melin-Norkrans medium and ¼ PDA medium. A sterile slide was then diagonally inserted into the culture medium and incubated in a biochemical incubator (SPX-70BX, Tianjin Instrument Co., LTD, China) at 27°C in the dark for 5 days (Arifin et al., 2020). Once the slide was covered with growth from the BJ1 strain, it was stained with Hoechst 33342. The Hoechst 33342 solution was prepared according to the protocol reported by Arifin et al. (2020), utilizing phosphate buffer solution at pH 7.8 to dissolve Hoechst 33342, which facilitating clearer observation of both the cell nucleus and septum of the strain. After staining with Hoechst 33342 solution in darkness for 20 min and rinsing with sterile water, samples were scanned using a fluorescence scanning analyzer (VS120-S6-W, OLYMPUS, Japan) at magnifications of ×40 and ×100. Finally, morphological identification of the fungi was conducted following guidelines outlined in the *Fungi Identification Handbook* (Wei, 1979).

### Molecular identification

According to the report by Ruibal et al. (2013), PCR amplification was conducted on four gene loci: the ribosomal internal transcribed spacer (ITS1-ITS4), glutamic synthetase (C4102), ATP synthetase (C14436), and ATP helicase (C3304), using the specific primers and PCR protocols detailed in **Table 1**. The PCR products were subsequently separated and purified via agarose gel electrophoresis and submitted to Shanghai Sangon Biotechnology Co., Ltd. for sequencing. The obtained sequences were analyzed using MEGA v.7 software. The evolutionary history was inferred by using the Maximum Likelihood method based on the Kimura 2-parameter model. Initial tree(s) for the heuristic search were obtained by applying Neighbor-Join and BioNJ algorithms to a matrix of pairwise distances estimated using the Maximum Composite Likelihood (MCL) approach, and then selecting the topology with superior log likelihood value. The analysis involved 22 nucleotide sequences. Codon positions included were 1st+2nd+3rd+Noncoding. All positions containing gaps and missing data were eliminated. There were a total of 535 positions in the final dataset.

**Table 1:** Primers and PCR conditions.

Primers	Forward	Reverse	PCR condition
ITS (ITS1-ITS4)	5’-TCCGTAGGTGAACCTGCGG-3’	5’-TCCTCCGCTTATTGATATGC-3’	An initial denaturation at 95°C, for 2 min, followed by 39 PCR cycles (denaturation at 95°C for 1 min; annealing at 50°C for 1 min; extension at 72°C for 1 min) before a final extension at 72°C for 10 min
ATP mitochondrial (C14436F, C14436R)	5’-ATGGACGGTACYGADGGTCTYG-3’	5-CACGGAAGTAYTCNGCRATGG-3′	94°C for 3 min; 12 cycles (touchdown) of 94°C for 30 s, 66°C for 40 s (−3°C/s cycle), and 72°C for 1 min; 30 cycles of 94°C for 30 s, 48°C for 40 s, and 72°C for 1 min; 72°C for 20 min; and 11°C on hold
Glutamate synthase (C4102F, C4102R)	5’-ATCAARTAYGCTGGCCTKCCTTGG-3’	5’-CGRCCGCCWGTCATGTACTCGCA-3’	94°C for 3 min; 12 cycles (touchdown) of 94°C for 30 s, 66°C for 40 s (−3°C/s cycle), and 72°C for 1 min; 30 cycles of 94°C for 30 s, 48°C for 40 s, and 72°C for 1 min; 72°C for 20 min; and 11c on hold
ATP mitochondria C3304	5’-TTGAAGTCACCGGGAAGAAC -3’	5’-CGGCGTTACGCTTSGTCT-3’	A 3-min denaturation at 94°C; followed by 12 touchdown cycles at 94°C (30s), 66°C (40 s) (−3°C/second cycle), 72°C (1 min); 30 cycles at 94°C (30s), 48°C (40s),72°C (1 min); and final extension at 72°C

#### Hydrolytic enzyme production by BJ1

BJ1 was cultured on Potato Dextrose Agar (PDA) for 5 to 8 days. Subsequently, the culture was punched in the medium using the end of a sterile gun head (φ = 10 mm) and inoculated at the center of various screening media: PDA-ABTS (2, 2’-diazo-bis-3-ethylbenzothiazolin-6- sulfonic acid) screening medium (ABTS 0.03%, KNO_3_ 3 g/L in PDA medium, sterilized at 121°C for 20 min), pectinase screening medium (NH_4_Cl 0.4g/L, K_2_HPO_4_ 1 g/L, MgSO_4_·7H_2_O 2 g/L, FeSO_4_·7H_2_O 0.01 g/L, pectin 8 g/L, agar 20 g/L; pH 7, sterilized at 121°C for 20 min), and protease screening medium (skimmed milk powder 75 g/L, agar 15 g/L, sterilized at 110°C for 10 min) respectively (Zhu, 2018; Chi et al., 2022; Zhou et al., 2020). Each treatment group was set up with three biological replicates and incubated in a biochemical incubator at 25°C in the dark for six days. The enzymatic activity was evaluated based on the presence of a clear zone around these colonies. The enzymatic index (EI) was calculated using the formula EI = diameter (mm) of the discolored halo/diameter (mm) of the colonies (DeFátima et al., 2021).

#### Phosphorus-solubilizing activity of BJ1

BJ1 was inoculated onto Monkina phosphorus-free medium, which consisted of C_6_H_12_O_6_ 10.00 g/L, (NH_4_)_2_SO_4_ 0.50 g/L, NaCl 0.30 g/L, KCl 0.30 g/L, FeSO_4_·7H_2_O 0.03 g/L, MnSO_4_·4H_2_O 0.03 g/L, MgSO_4_·7H_2_O 0.30 g/L, Ca_3_(PO_4_) _2_ 5.00 g/L, agar 20.00 g/L, and water up to 1000 mL; the pH was maintained between 7.00 and 7.50, with sterilization conducted at 115°C for 30 min. Additionally, BJ1 was cultured on organic phosphorus solid medium prepared by cooling 1000 mL of beef extract peptone medium to below 50°C, followed by the immediate addition of 60 mL sterile saline and egg yolk liquid in a ratio of 1:1 before pouring into plates. Observation of a clear halo surrounding the colonies indicated that the strain exhibited phosphate-solubilizing activity (Li et al., 2021; Chen et al., 2009; Xue et al., 2019).

#### Siderophore production of BJ1

BJ1 was cultured at 25°C for a period of 7 days, after which it was transferred to CAS medium. The composition of the CAS medium included: 60.5 mg of CAS, 72.9 mg of cetyltrimethyl ammonium bromide (HDTMA), 10 mL of a 1 mmol/L FeCl_3_·6H_2_O (dissolved in 10 mmol/L HCl), along with 50 mL of a 0.1 mol/L phosphate buffer, 9.0 g of agar, and deionized water to make up a total volume of 1000 mL (Xu et al., 2022). The appearance of an orange siderophore chelate indicates that the strain exhibits siderophore-producing activity.

#### Plant growth hormone production ability of BJ1

##### Salkowski test

The BJ1 strain was inoculated into PDA liquid medium, either supplemented with L-tryptophan (100 mg/L) or without supplementation. Cultures were incubated at 25°C on a shaker (SKY-211B, Jingda Instrument manufacturing Co., LTD, China) set to 200 rpm for 5 days. Following incubation, the cultures were centrifuged at 12,000 rpm for 2 min. The supernatants were then mixed in a 1:1 ratio with Salkowski reagent (50 mL, 35% of perchloric acid, 1 mL 0.5 M FeCl_3_ solution) and transferred to a colorimetric cup for observation of any color changes. The presence of indole-3-acetic acid (IAA) or similar compounds in the supernatant will result in the development of a pink color upon reaction with Salkowski reagent (Wang et al., 2020; Zhang et al., 2016). A darker hue indicates a higher secretion capacity (Patel et al., 2018).

##### IAA content determination

Mycelium blocks (9 mm in diameter) of the BJ1 strain, cultivated on PDA for 5 days were transferred into 200 mL of PDA liquid supplemented with 100 mg/L L-tryptophan and cultured at 25°C and agitated at 200 rpm. The fermentation supernatant and mycelium were subsequently collected, and the IAA secretion capability of BJ1 strain was evaluated using fermentation broth from the BJ1 strain without L-tryptophan as a control (Zhang et al., 1999; Cheng et al., 2022).

(Extraction of fermentation broth) A total of 500 mL each of BJ1 fermentation broth containing L-tryptophan and that not containing L-tryptophan was transferred to a round-bottom flask and concentrated to 35 mL under reduced pressure at 50°C. Then, 105 mL of absolute ethanol was added to make a final concentration of ethanol of approximately 75%. After filtration and concentration, the residues were dissolved in 75% ethanol to 5 mL and filtered using a 0.22 μm Millipore filter (Merck Millipore, Germany) for High Performance Liquid Chromatography (HPLC) analysis.

(Extraction of mycelial contents) The hyphae were dried, ground into powder, weighed (6.0 g), and refluxed with 100 mL of 75% ethanol for two hours. A portion measuring five milliliters from this mycelium extract was taken for Salkowski detection. The extracts were concentrated under reduced pressure and dissolved with 75% ethanol to 5 mL, and then filtered through a 0.22 μm Millipore filter for HPLC analysis.

Gibberellin (GA3), IAA, abscisic acid (ABA), 1-naphthylacetic acid (NAA), 6-benzylaminopurine (6-BA), and kinetin (KT) were accurately weighed and dissolved in 75% ethanol to prepare a standard solution, each with a concentration of 1 mg/mL. The IAA stock solution was diluted with 75% ethanol to prepare a series of IAA standard solutions, which were then injected into the HPLC system for the construction of the standard curve (Wang et al., 2018; Shah et al., 2021). Samples were injected into an UltiMate3000 HPLC system (Diane, California, USA) coupled with a GL SCIENCES Inertsil ODS-EP C18 column (4.6 × 250 mm, 5 μm), and gradient elution with acetonitrile and 0.1% formic acid aqueous solution at a flow rate of 1 mL/min as detailed in **Table 2**. Detection was carried out at a wavelength of 270 nm (Wu, 2019).

**Table 2:** Chromatographic conditions.

T (min)	A (acetonitrile)	B (0.1% formic acidaqueous solution)
0	5	95
40	95	5
45	95	5
55	95	5

#### Plant growth-promoting assay and endophytic colonization

Fully mature *B. striata* fruit clips were selected for measurement. The seeds of *B. striata* were found to be approximately 1 to 2 millimeters in length and 0.2 millimeters in width, with a thousand-seed weight ranging from 1~2×10^-3^ g. The fruit clips underwent disinfection through a sequential treatment involving immersion in 75% ethanol for 5 minutes, followed by exposure to a solution of 4% sodium hypochlorite for an additional 7 minutes; they were then rinsed five times with sterile water. The fruit clip was then dissected, and the seeds were suspended in a 1% agar solution. Subsequently, approximately 100 seeds were evenly dispersed on agar medium for symbiotic cultivation, with non-symbiotic cultivation serving as the control. Seeds were cultured in an artificial climate chamber (LISK Instrument Equipment Co., LTD, NanJing, China) at a temperature of 25°C under conditions of 12 h light and 12 h darkness. The germination rate of *B. striata* seeds was monitored weekly over a four-week period according to criteria specified in previous publications (Stewart and Zettler, 2002; Tian et al., 2023). The stages of seed germination and seedling development in Orchidaceae plants are classified as follows: stage 0 indicates no germination; stage 1 involves rupture of the testa due to embryo expansion; stage 2 marks the formation of protocorms through spherical expansion of the embryo; stage 3 is characterized by the appearance of protomeristems; stage 4 denotes leaf emergence; and stage 5 signifies leaf elongation leading to seedling formation. Furthermore, from each treatment group, three sets of protocorms were randomly collected on a weekly basis, with each set consisting of 15 protocorms. Their length, width, and fresh weight were measured using the method described by Yamamoto (Yamamoto et al., 2017).

Symbiotic and non-symbiotic protocorms were randomly selected to observe fungal colonization. The protocorms were transferred to 1.5 mL Eppendorf tubes, treated with a 10% KOH solution by heating in a water bath at 90°C for 8 hours. Following this, the KOH solution was removed, and the protocorms were washed three times. They were then sequentially soaked in 10% hydrogen peroxide solution and 5% acetic acid for 1 h each. Finally, the protocorms were stained with a 0.04% Trypan blue solution for 10 min. Fungal colonization was observed with a microscope (Jiang et al., 2019).

#### Synergistic effect of L-tryptophan and BJ1 on the growth of *B. striata* seeds

*B. striata* seeds were evenly distributed across four distinct types of 1.5% agar medium, which included symbiotic agar media both with and without the addition of 100 mg/L L-tryptophan, as well as non-symbiotic agar media under the same conditions. The cultures were incubated in an artificial climate chamber maintained at 25°C with a photoperiod of 12 h light and 12 h darkness. Fifteen *B. striata* protocorms were collected every 7 days. The fresh weight, width, and length of *B. striata* seeds were determined. The synergistic effect of L-tryptophan in conjunction with BJ1 on the growth rate of *B. striata* seeds was evaluated using the growth rate model G = (a − b)/b × 100% (Yamamoto et al., 2017; Jahn et al., 2021).

### Statistical analysis

GraphPad Prism 8.0 (GraphPad Software, La Jolla, CA, USA) was utilized for data processing, statistical analysis, and graphical representation. All data are expressed as mean ± standard deviation. One-way ANOVA was employed to compare differences between groups, followed by Tukey’s test for multiple comparisons. An independent-samples *t*-test was used to analyze the differences between the two groups. *p* < 0.05 was considered statistically significant.

## Results

### Morphological and molecular biological identification of BJ1

**Figure 2:**
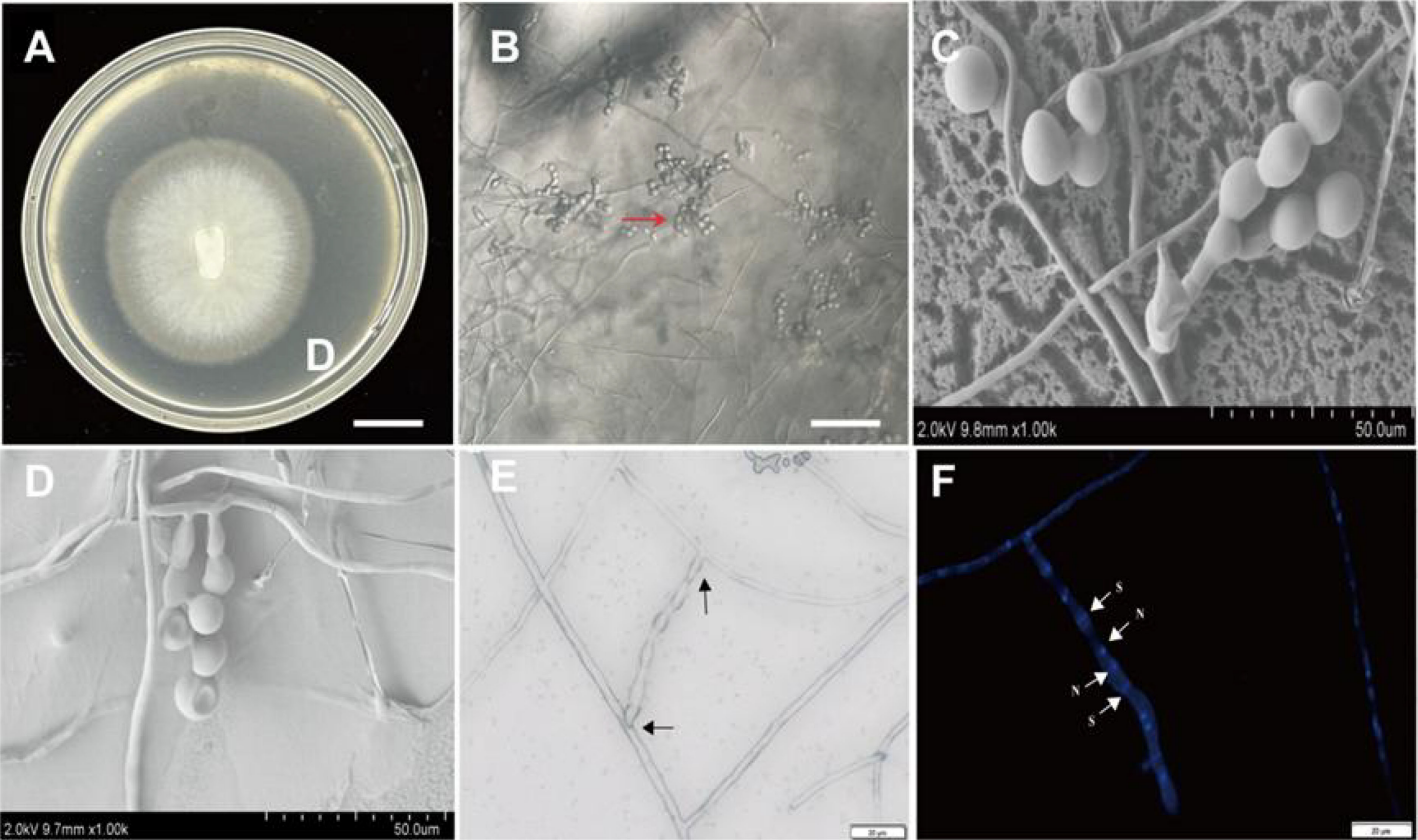
Morphological characteristics of BJ1. **(A)** Colony morphology of BJ1 cultured for 8 days (bar = 1 cm); **(B)** morphology of mycelium and monilioid cells under a microscope (bar =100 μm); **(C)** Morphology of hyphae and monilioid cells under a scanning electron microscope (bar = 50 μm); **(D)** mycelium interweaving and branching at right angles under a scanning electron microscope (bar = 50 μm); **(E)** The morphology of the hyphae under visible light, showing with branching at right angles, cylindrical hyphae, with cylindrical terminal elements (black arrow) bars = 50 µm; **(F)** The morphology of hyphae stained with Hoechst 33342, showing binucleate cells (white arrow, N = nuclei; S = septa);. Bars = 20 µm.

After an incubation period of 8 days at 25°C, the BJ1 strain exhibited the development of white to cream-colored colonies on PDA, characterized by concentric rings and undulated, submerged edges. The aerial mycelium was not well-developed (**Figure 2A**). Microscopic observation showed that BJ1 could produce elliptical monilioidcells at the apex of the mycelia (**Figure 2B**), a finding further corroborated by scanning electron microscopy (**Figures 2C, D**). In addition, many mycelia frequently formed clusters and produced lateral branches at typical right angles or acute angles (**Figures 2E, F**) (Freitas et al., 2020; Linde et al., 2017). Scanning electron microscopy revealed that the monilioid cells of the BJ1 strain exhibited significant morphological variability, typically appearing spherical or ellipsoidal with diameters ranging from 5 to 10 µm. These cells formed chain-like structures, which could be branched, with each branch usually comprising 2 to 5 monilioid cells. The nuclear staining indicates binuclear cells (**Figure 2F**). There are structural similarities between the morphological observations and those described for *Tulasnella* sp.

**Figure 3:**
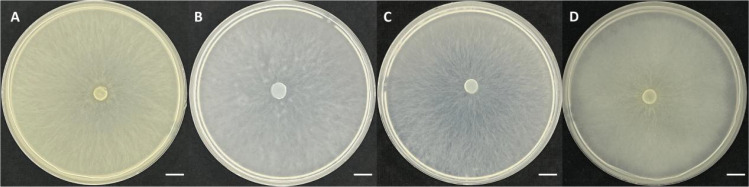
The morphology of strain BJ1 cultured on **(A)** ¼ PDA, **(B)** ½ FIM, **(C)** 3MN + vitamin, and **(D)** E-medium. Bars = 1cm.

According to the culture medium types used in reported articles (½ FIM medium, E-medium, 3MN/Magali’s Modified Melin-Norkrans medium and ¼ PDA medium) and the same medium conditions, BJ1 was cultured in the same way (Arifin et al., 2020; Linde et al., 2017; Ruibal et al., 2013). The results showed that after the same culture for 4 weeks, BJ1 hyphae had completely covered the entire medium, and the hyphae growth rate was faster than that of the known *Tulasnella* mycorrhizal fungus. The aerial hyphae of the BJ1 strain are more developed; it is quite different from the reported new *Tulasnella* mycorrhizal fungi (**Figure 3**). Nevertheless, a more rigorous and systematic comparison of the related strains under standardized conditions is essential to elucidate their differences in growth characteristics.

**Figure 4:**
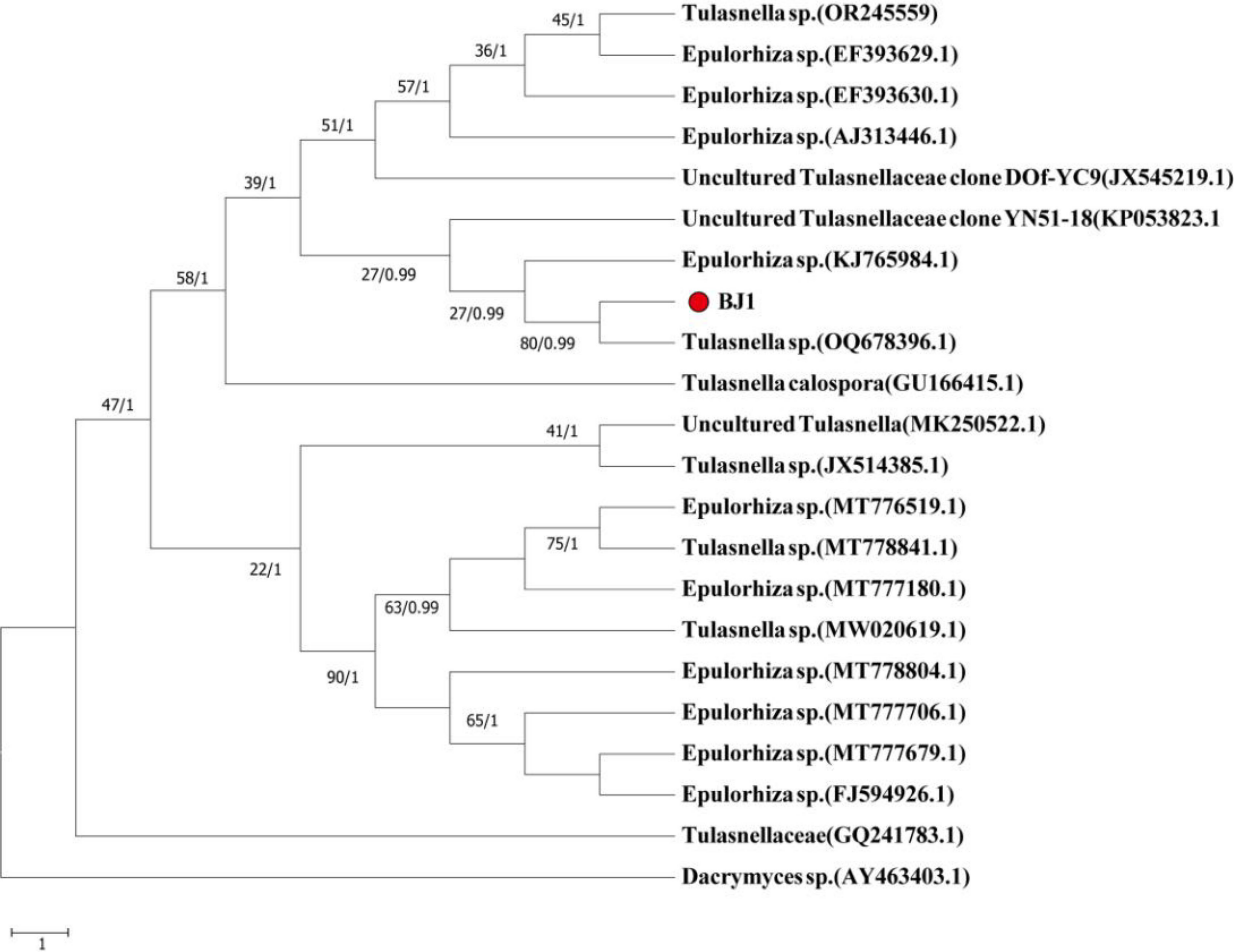
Construction of phylogenetic tree based on ITS sequence analysis results.

Molecular identification indicated that BJ1 (OR186273.1) and *Tulasnella* sp. (OQ678396.1) clustered within the same phylogenetic branch. A comparison of their ITS sequences showed a query cover of 93%, with a similarity percentage of only 90.56% (**Figure 4**). The BJ1 strain was still branching independently, according to the results of the phylogenetic tree that was built using the other loci (C14436, C4102, and C3304). All three loci had high bootstrap (>90) and BPP values (**Supplementary Figures S2-S4**). Consequently, the strain BJ1 is likely representative of a novel species within the *Tulasnella* genus that has not been previously documented.

### Analysis of hydrolytic enzymes produced by BJ1

**Figure 5:**
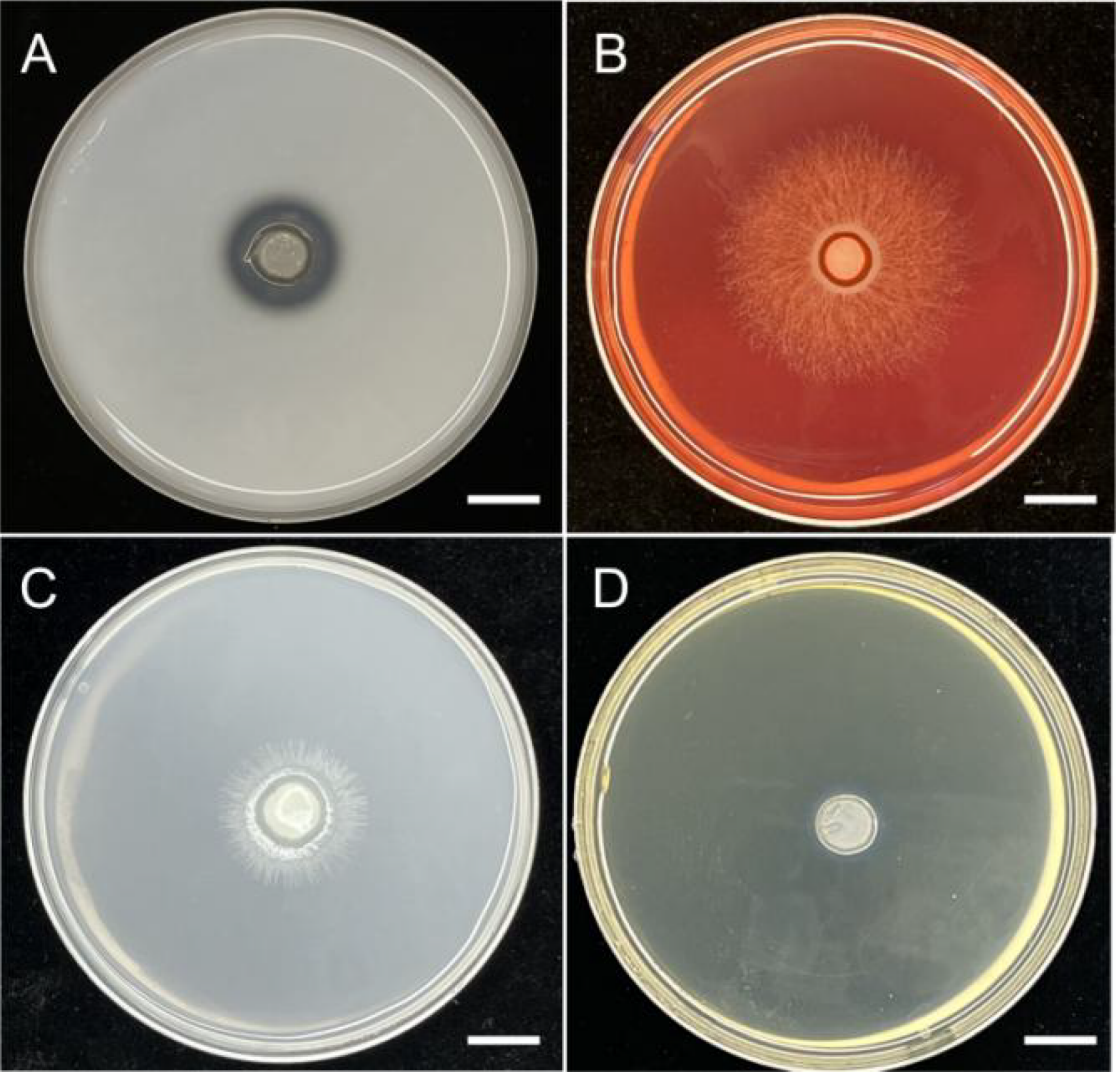
Enzyme activity analysis of BJ1 strain. Transparent circles appear on the culture medium in primary screening of **(A)** protease, **(B)** pectinase, **(C)** inorganic phosphate-solubilizing activity, and **(D)** siderophore-producing activity (bar = 1 cm).

The results demonstrated that BJ1 could secrete extracellular enzymes, including pectinase and protease (**Figures 5A, B**), with respective activity indices of 0.30 ± 0.05 and 1.32 ± 0.08. However, the absence of a discernible transparent circle on the PDA-ABTS screening medium suggests that BJ1 may have a limited ability to secrete laccase.

### Analysis of phosphorus solubilization and siderophore production of BJ1

The strain inoculated on Montina inorganic phosphorus medium was distinctly surrounded by a transparent circle (**Figure 5C**), indicating that the BJ1 strain possesses a certain capacity to dissolve inorganic phosphorus, with an enzyme index of 0.59 ± 0.03. However, no noticeable transparent circle was observed around the strain on the organic phosphorus solid medium, implying that the BJ1 strain may not be capable of dissolving organic phosphorus (**Table 3**). In addition, when the BJ1 strain was inoculated onto the CAS plate, it did not produce yellow siderophore, indicating that BJ1 lacks the ability to produce siderophore (**Figure 5D**).

**Table 3:** Results of extracellular enzyme and phosphorus dissolving activity of BJ1.

	Incubating time (day)	Colony diameter (mm)	Transparent ring diameter (mm)	EI (Enzyme Index)	Color description
Laccase	6	63.00 ± 4.00	/	/	/
Protease	6	15.53 ± 1.03	20.53 ± 2.57	1.32 ± 0.08	Transparent and colorless
Pectinase	6	42.63 ± 2.47	12.93 ± 1.16	0.30 ± 0.05	Transparent and colorless
Inorganic phosphorus	3	25.06 ± 1.52	14.7 ± 1.36	0.587 ± 0.03	Transparent and colorless
Organophosphorus	3	16.40 ± 1.57	/	/	/

### IAA production by BJ1

**Figure 6:**
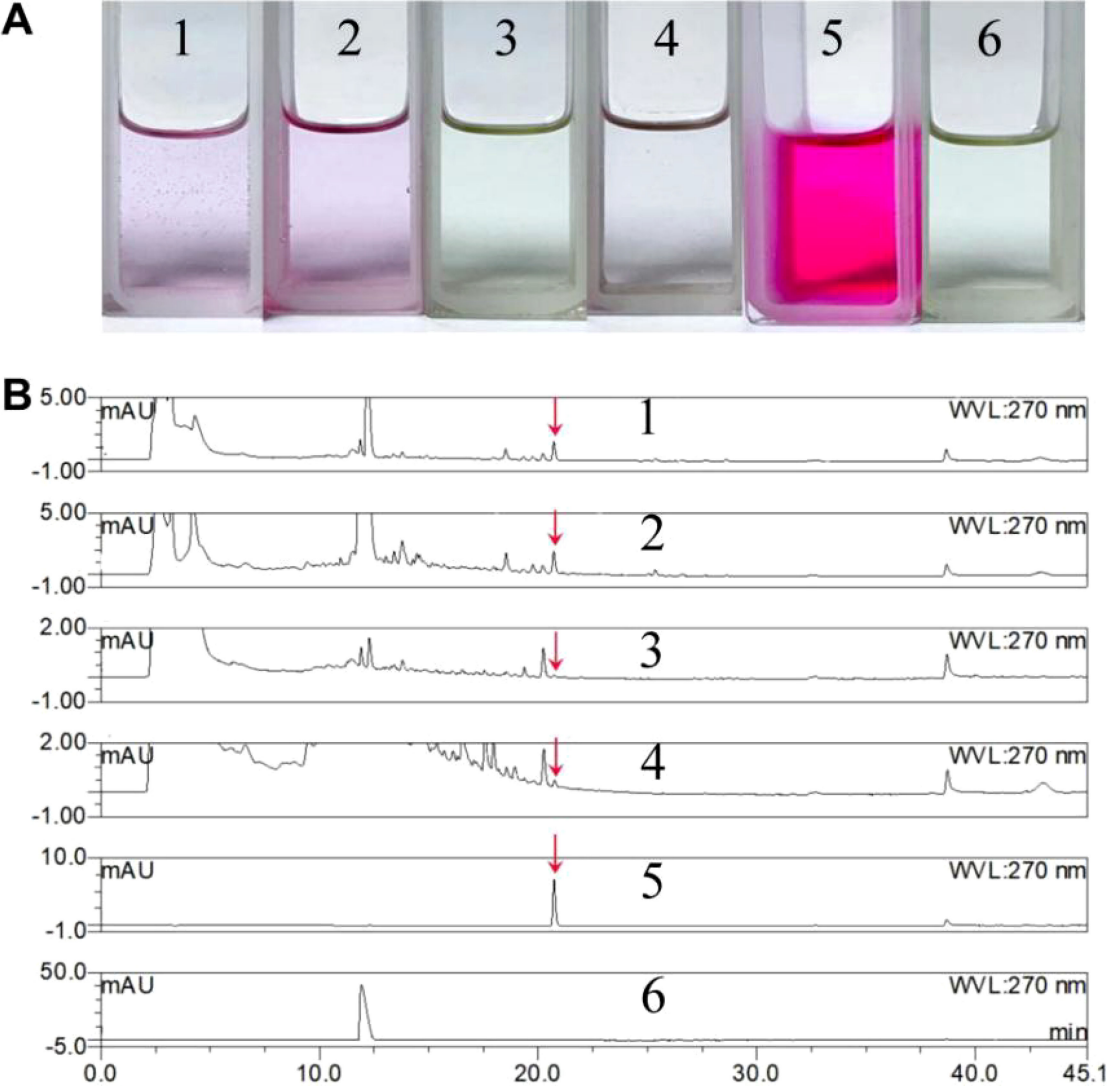
Qualitative and quantitative analysis of IAA. **(A)** Salkowski colorimetry; **(B)** HPLC analysis. 1 BJ1 fermentation (with L-tryptophan) broth extract, 2 BJ1 fermentation (with L-tryptophan) mycelia extract, 3 BJ1 fermentation (without L-tryptophan) broth extract, 4 BJ1 fermentation (without L-tryptophan) mycelia extract, 5 the standard of IAA, and 6 the standard of L-tryptophan. The red arrow indicates the peak of IAA.

The secretion of growth hormone represents one molecular mechanism through which mycorrhizal fungi facilitate the growth and germination of orchid seeds (Patel et al., 2018; Wu et al., 2002). The results indicated that the unfermented control PDA liquid medium, regardless of whether L-tryptophan was present, did not exhibit a significant color reaction; In contrast, both the fermentation supernatant and mycelial extract from BJ1 cultured in PDA liquid medium supplemented with L-tryptophan displayed a distinct pale pink coloration. Surprisingly, neither the BJ1 fermentation broth nor the mycelia extract of PDA liquid culture without L-tryptophan showed any notable color change (**Figure 6A**). These findings indicated that strain BJ1 is capable of synthesizing IAA when L-tryptophan serves as a substrate; however, no positive reaction was detected in the absence of L-tryptophan, which may be attributed to the low content of IAA. Furthermore, HPLC analysis was performed to analyze if BJ1 might produce other types of plant hormones aside from IAA. As depicted in **Supplementary Figure S5**, no additional hormones were detected in the concentrated extract of BJ1 fermentation broth apart from IAA. Thus, we further analyzed IAA production under varying fermentation conditions. Remarkable IAA peaks were detected in both the culture supernatant and mycelium extracts derived from BJ1 fermentation with L-tryptophan (**Figure 6B**, 1 and 2). In addition, traces of IAA were also found in both the fermentation supernatant and mycelium extract without L-tryptophan (**Figure 6B**, 3 and 4), but the content was significantly lower than those obtained with L-tryptophan supplementation. The content of IAA under different culture conditions was also quantified based on a standard curve: *Y* = 14.90*X* − 0.076, R^2^ = 0.999. The concentrations measured for IAA in fermentation broths with and without L-tryptophan were determined to be 0.0217 mg/mL and 0.0076 mg/mL, respectively; similarly, for dried mycelia they were recorded at concentrations of 0.0188 mg/g and 0.0061 mg/g, respectively.

### BJ1 endophytic colonization and growth promotion of *B. striata* seeds

**Figure 7:**
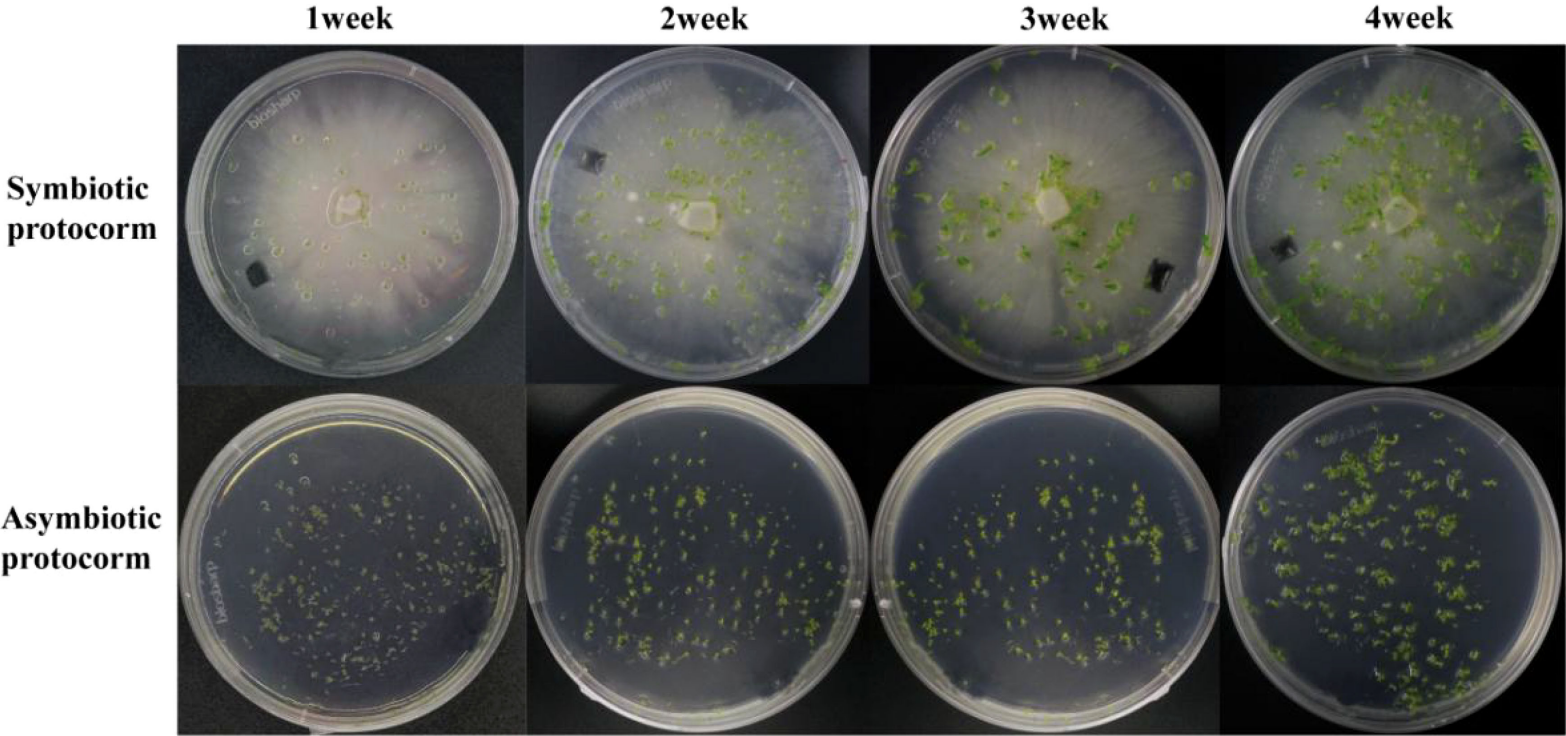
Germination and growth of symbiotic and non-symbiotic *B striata* seeds.

According to the criteria established by Stewart and Zettler (2002) for the germination and growth of Orchidaceae plants, we calculated the symbiotic germination rates of mycorrhizal fungi BJ1 and *B. striata* seeds at different time intervals (**Figure 7**). The results showed that mycorrhizal fungus BJ1 significantly promoted the germination of *B. striata* seeds. The germination rate in the symbiotic group was notably higher than that in the control group (**Table 4**). In the first week, both groups reached stage 2 with percentages of 71.32 ± 8.12% for the symbiotic group and 71.17 ± 8.15% for the control group, showing no significant difference between them. However, only the symbiotic group reached stage 3 at a rate of 12.42 ± 4.11%, while no seeds from the non-symbiotic group reach this stage. Similarly, during weeks 2 and 3 of cultivation, growth in the symbiotic group outpaced that in the control group, with percentages reaching stages 4 and 5 at rates of 16.16 ± 4.25% and 15.22 ± 3.49%, respectively. By week 4, a significantly higher percentage of seeds in the symbiotic group had attained stage 5 (74.23 ± 11.21%) compared to those in the control group (53.23 ± 11.35%). Significant differences were observed between these two groups.

**Table 4:** Effect of mycorrhizal fungus BJ1 on seed germination rate of *B. striata*.

	Symbiotic protocorm				Asymbiotic protocorm			
	1 week (%)	2 week (%)	3 week (%)	4 week (%)	1 week (%)	2 week (%)	3 week (%)	4 week (%)
GR	79.18 ± 0.71	93.92 ± 3.08	93.90 ± 3.09	96.07 ± 2.88	71.18 ± 1.42^*^	89.89 ± 2.95	92.48 ± 2.55	93.04 ± 1.45^*^
Stage 1	21.12 ± 1.13	6.43 ± 3.21	7.11 ± 3.21	4.22 ± 3.11	29.33 ± 8.16	10.26 ± 3.21	8.11 ± 3.29	7.01 ± 1.01^**^
Stage 2	71.32 ± 8.12	3.25 ± 2.47	5.44 ± 1.45	0.00 ± 0.00	71.17 ± 8.15	27.38 ± 11.22^**^	6.11 ± 7.31	0.00 ± 0.00
Stage 3	12.42 ± 4.11	75.19 ± 5.48	16.21 ± 6.33	4.29 ± 2.33	0.00 ± 0.00^**^	63.45 ± 13.26^*^	31.22 ± 10.16^*^	3.11 ± 3.03
Stage 4	0.00 ± 0.00	16.16 ± 4,25	62.27 ± 14.44	19.01 ± 10.20	0.00 ± 0.00	0.00 ± 0.00^**^	55.46 ± 11.28	37.15 ± 11.23
Stage 5	0.00 ± 0.00	0.00 ± 0.00	15.22 ± 3.49	74.23 ± 11.21	0.00 ± 0.00	0.00 ± 0.00	0.00 ± 0.00^**^	53.43 ± 11.35^*^

GR, Germination rate. Compared to the corresponding weekly symbiosis group, there was a statistically significant difference, ^*^ *p* < 0.05; ^**^ *p* < 0.01.

**Figure 8:**
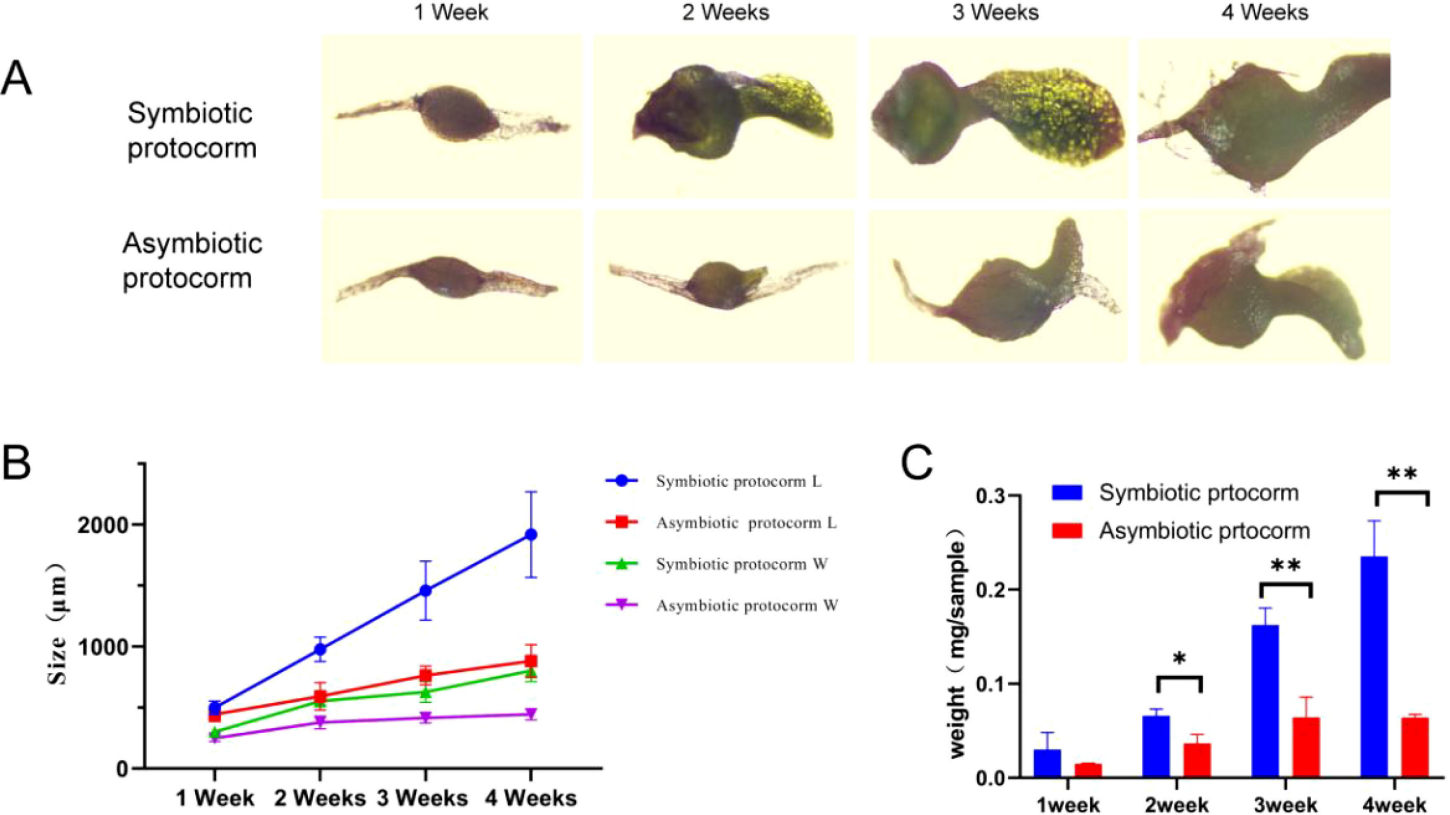
Effect of BJ1 symbiosis on the growth of *B striata* protocorm. **(A)** Effects of symbiosis and non-symbiosis culture on the growth of *B striata* protocorms. Scale = 500 μm; **(B)** length and width changes of *B striata* protocorms under symbiotic and non-symbiotic conditions. Solid circles and triangles represent the length (L) and width (W) of the symbiotic group, respectively. Rectangles and inverted triangles represent the length (L) and width (W) of non-symbiotic groups, respectively; **(C)** weight of symbiotic and non-symbiotic *B striata* protocorms. The error line represents the standard error of the average value of 15 protocorms. Independent-samples *t*-tests were used to determine the statistically significant differences in seed weight: ^*^ *p* < 0.05, ^**^ *p* < 0.01.

Furthermore, we conducted weekly comparisons regarding growth morphology, size, and weight among representative seeds from each treatment group (**Figure 8A**). After 1 week of culture, *B. striata* seeds began absorbing water and expanding into rounder shapes; however, there was no significant difference noted between the symbiotic group and non-symbiotic group. After 2–4 weeks of culture, the disparity in length and width between both groups progressively increased. The symbiotic group showed incremental growth with a higher growth rate, whereas the non-symbiotic group showed steady growth with a lower growth rate. This may be attributed to the symbiotic relationship between strain BJ1 and *B. striata* seeds, which accelerates its nutrient supply and plant hormone secretion, thereby promoting the rapid growth of the *B. striata* protocorm (**Figure 8B**). After 2–4 weeks of culture, significant statistical differences in protocorm weight were also noted between the symbiotic and non-symbiotic groups (**Figure 8C**).

**Figure 9:**
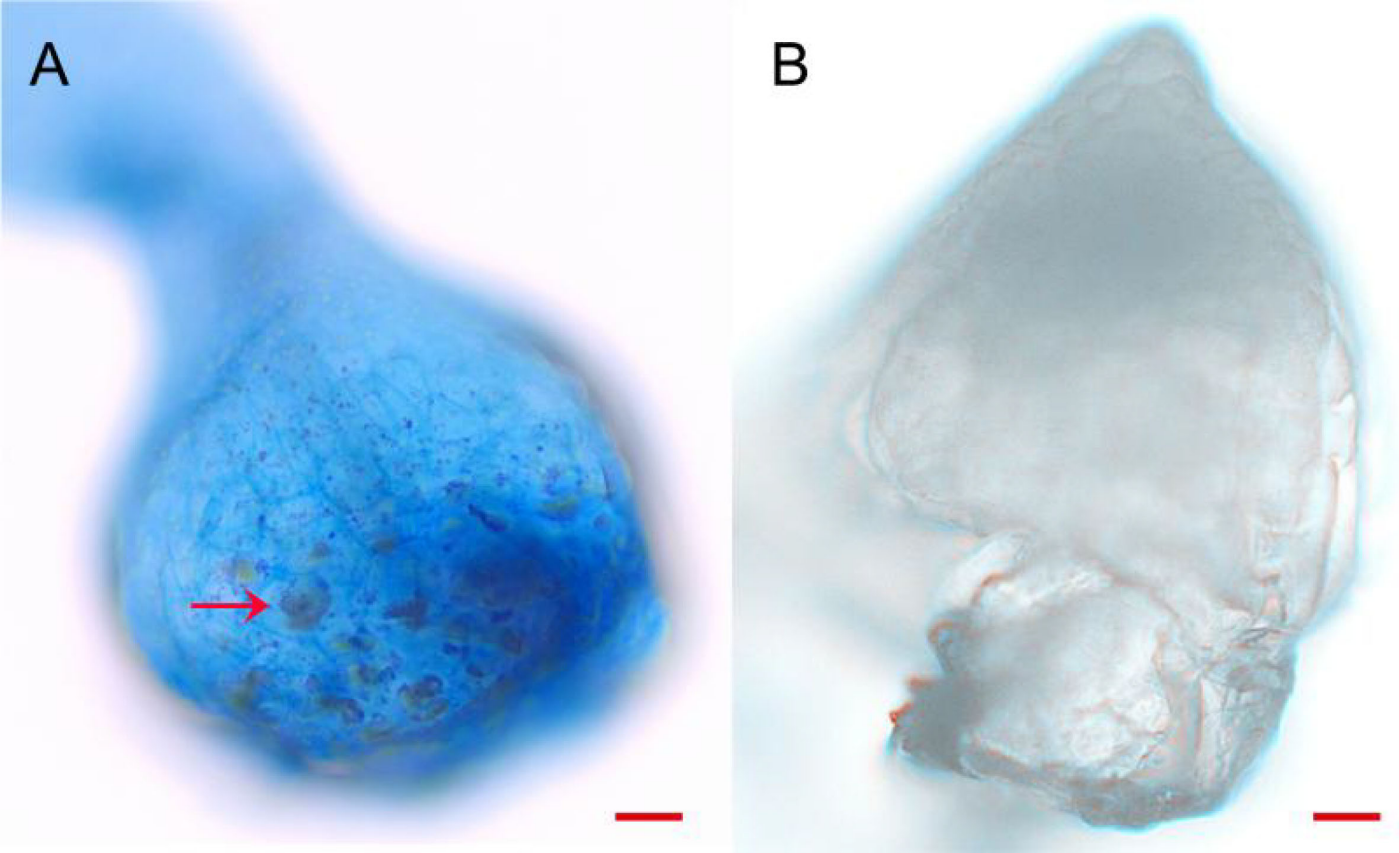
Trypan blue staining of endophytic colonization. **(A)** Endophytic colonization in protocorm of *B striata* (arrow) (bar = 50 μm); **(B)** protocorm of *B. striata* without BJ1 symbiosis (bar = 50 μm).

Trypan blue staining demonstrated that the strain BJ1 primarily colonized at the base of the protocorm (**Figure 9A**); conversely, no mycelia clusters were observed in the control group (**Figure 9B**). This observation aligns with findings reported by Jiang (Jiang et al., 2019).

### Synergistic effect of BJ1 and L-tryptophan on the growth of *B. striata* seeds

**Figure 10:**
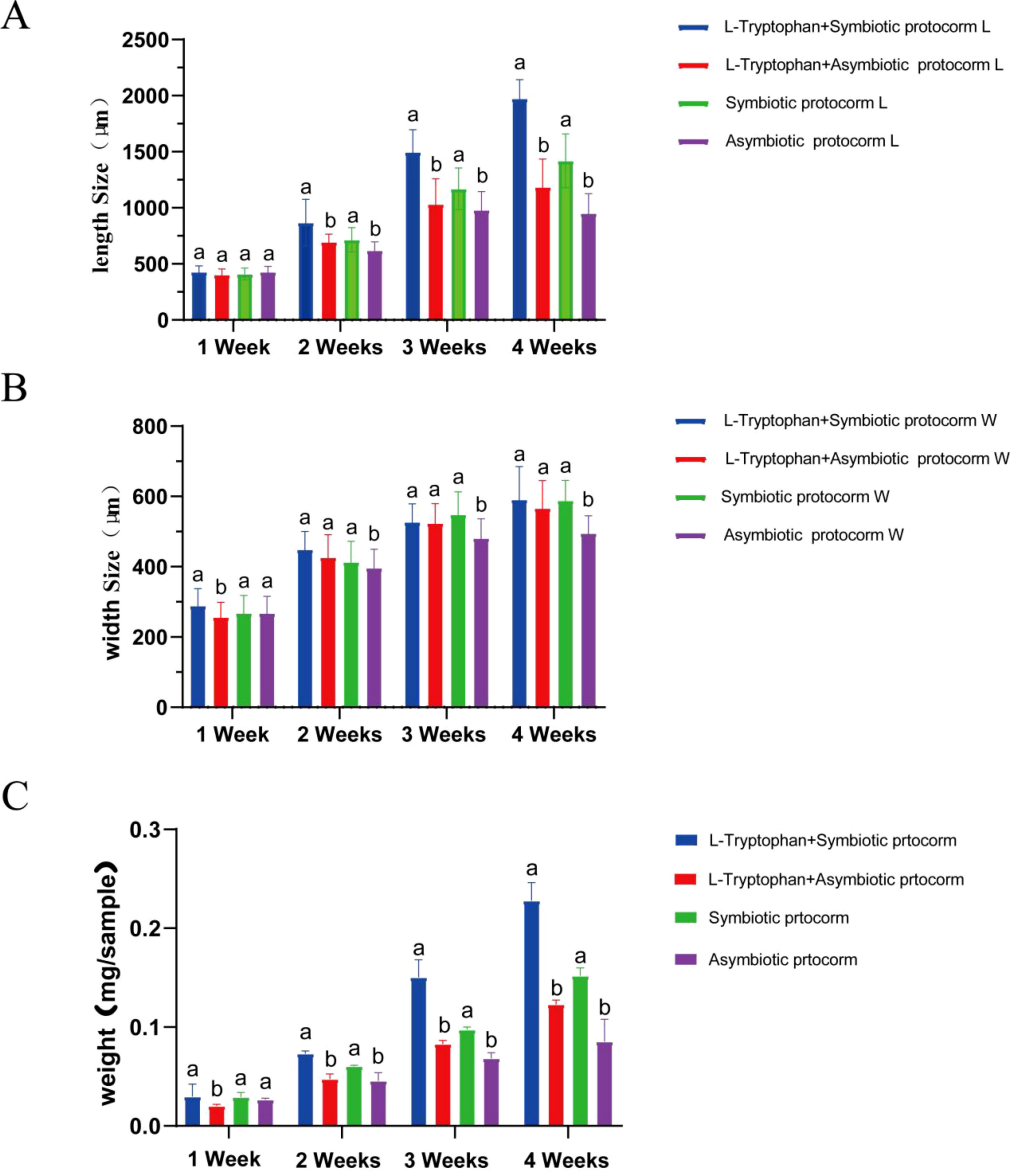
BJ1 and L-tryptophan synergistically promote the growth of *B striata* seeds. Changes in protocorm **(A)** length, **(B)** width, and **(C)** weight of *B striata* under different culture conditions. Standard error of the average value of 15 protocorms. One-way ANOVA was used for comparison between groups followed by Tukey’s test for multiple comparisons. Different letters indicate significant differences between groups in the same week, *p* < 0.05.

BJ1 can utilize L-tryptophan as a substrate to promote IAA synthesis; thus, we investigated whether the combination of L-tryptophan and BJ1 could synergistically enhance the growth of *B. striata* seeds. As presented in **Figure 10**, the length, width, and weight of the *B. striata* protocorm in the control group (without BJ1 and L-tryptophan treatment) gradually increased in the second and third weeks, but no significant growth was observed in the fourth week. Both L-tryptophan and BJ1 effectively promoted the growth of the *B. striata* protocorms after co-culturing with either L-tryptophan or BJ1 for 4 weeks, with BJ1 demonstrating superior efficacy compared to L-tryptophan. When seeds were co-cultured with both L-tryptophan and BJ1 for 4 weeks, their effects on both length and weight of the *B. striata* protocorms were significantly greater than those observed when seeds were co-cultured solely with either L-tryptophan or BJ1 individually. In particular, the weight of fresh protocorms co-cultured with L-tryptophan and BJ1 was 2.6 times that of the control, even heavier than the total weight of protocorms co-cultured with L-tryptophan or BJ1 alone, with the growth rate for weight reaching 155.32%. Collectively, these findings support the notion that L-tryptophan and BJ1 can work synergistically to promote both growth and development in *B. striata* seeds.

## Discussion

It is widely recognized that mycorrhizal fungi are essential for the germination and growth of orchid seed in their natural environments (McCormick et al., 2018). Hundreds of fungal species have been isolated and identified from various orchid species, including those belonging to *Basidiomycota* and *Deuteromycota*. The most prevalent orchid mycorrhizal fungus is Rhizoctonia sensu lato, which encompasses three asexual genera: *Ceratorhiza*, *Epulorhiza*, and *Moniliopsis*. Additionally, these strains represent the predominant groups that significantly facilitate orchid seed germination and growth (Watkinson and Winkel, 2024). Further investigation into the biological characteristics of these mycorrhizal fungi and their symbiotic mechanisms with orchid plants can provide a robust theoretical foundation for optimizing seedling cultivation techniques and achieving sustainable green planting practices. Additionally, such research will contribute significantly to the conservation of orchid germplasm resources. In this study, we investigated the biological properties of *Tulasnella* sp. mycorrhizal fungus BJ1, which was isolated from the fibrous roots of *B. striata*, along with its role in promoting germination and growth of *B. striata* seeds.

In 1888, Schroter made the first discovery of the genus *Tulasnella*, which he named *Tulasnella lilacina* J. Schrot (Freitas et al., 2020). These fungi are found all over the world and mostly feed on decaying wood and liverwort or orchid mycobionts (Brundrett, 2002; Warcup, 1973). Through comprehensive research, it has been determined that *Tulasnella* sp. exhibits a lack of distinctive macromorphological characteristics, as well as a notable degree of morphological variation and overlapping dimensions among its microscopic components (Warcup, 1981). Therefore, distinguishing between different groups of *Tulasnella* sp. from a morphological perspective is challenging. In this study, the morphological characteristics of BJ1 strain were investigated. The results showed that there was no significant difference in morphological characteristics between BJ1 strain and reported *Tulasnella* sp fungi. However, when compared under the same medium conditions, the hyphae of BJ1 grew faster and the aerial hyphae were more developed (**Figure 3**). This indicated that BJ1 strains were different from other *Tulasnella* sp fungi. Since the reference strains mentioned in the literature were not available for direct comparison, further studies are required to confirm the observed differences in mycelial growth rate and colony morphology. The taxonomy of *Tulasnella* species presents considerable challenges owing to the ambiguous morphological and molecular characteristics they display. Cruz et al. (2014) propose an integrated approach that synthesizes molecular and morphological data for the identification of *Tulasnella* sp., thereby establishing a robust theoretical foundation for precise classification. Linde et al. (2017) used sequence data from 8 sequence loci (ITS, mtLSU, C4102, C14436, C3304, C12424, C4722, C10499) and coalescent-based species delimitation methods have revealed several species-level lineages of *Tulasnella* associated with the orchid in Australia. Arifin et al. (2020) identified new species of *Tulasnella* associated with Australian terrestrial orchids using 4 sequence loci (ITS, C14436, C4102, mtLSU). Due to the superior efficacy of the four sequence sites—the ribosomal internal transcribed spacer (ITS1-ITS4), glutamate synthase (C4102), ATP synthase (C14436), and ATP helicase (C3304)—in molecular identification of *Tulasnella* sp., this study selected these sequence sites for a more precise molecular identification of the BJ1 strain. The phylogenetic analysis revealed that the BJ1 strain maintained distinct independent branches (**Figure 4**; **Supplementary Figures S2–S4**). The low similarity to other *Tulasnella* species significantly enhances the probability that the BJ1 strain represents a novel strain.

According to the literature, mycelia invade the embryo cells from the base of the embryo before forming mycelia clusters within exodermal cells. These mycelia continue their invasion into plant cells for colonization; finally, dead mycelia will be digested and absorbed by the plant as nutrients to promote its growth. This process forms a symbiotic relationship with orchids. Trypan blue staining confirmed that the strain BJ1 primarily colonized at the base of the protocorm (**Figure 9A**), consistent with findings reported by Jiang et al. (2019). Mycorrhizal fungi often secrete catabolic enzymes such as protease and pectinase to degrade cell wall polymers (e.g., cellulose, xylan, and pectin), thereby facilitating invasion and parasitism (Kubicek et al., 2014). In addition, mycorrhizal fungi can promote the synthesis of plant secondary metabolites by secreting cell-degrading enzymes. They enhance plant stress resistance, facilitate nutrient absorption in plants, and contribute to ecological diversity and community stability (Fawad et al., 2024). In this study, we evaluated the enzyme activity of BJ1 and found that it exhibited comparable secretion capabilities of protease and pectinase to other *Tulasnella* sp. strains. However, while some of these fungi have strong secretion abilities, they may not necessarily promote seed germination (Tang, 2021). Some studies have reported that the fungus *Tulasnella* sp. may not have the ability to produce laccase (Tang, 2021), while others have shown that the fungus *Tulasnella* sp. can produce laccase but exhibits relatively low enzymatic activity (Zhao et al., 2021). However, strain BJ1 exhibited no detectable laccase activity (**Table 3**). Therefore, further investigation into the laccase production potential of *Tulasnella* sp. (BJ1) is warranted. Obviously, the symbiotic germination process is influenced by various factors beyond just the capacity to secrete cell-degrading enzymes. It is possible that multiple hydrolases function synergistically rather than contributing individually to this process or that the secretory capacity under pure culture conditions differs from that during a symbiotic relationship with seeds (Chen et al., 2022). Therefore, further investigation into the genetic basis of cell wall-degrading enzymes is warranted.

Plant growth requires the absorption of phosphorus and iron elements from the soil. Microorganisms that produce siderophore can fulfill plants’iron requirements, hasten the dissolution of iron ions in minerals, and facilitate the release of related elements from insoluble iron oxides (Ge et al., 2023). Phosphorus-solubilizing microorganisms enhance soil phosphorus availability, regulate soil conditions, and boost plant uptake and utilization of soil phosphorus. They also convert or activate otherwise inaccessible forms of phosphorus into available ones (Wang et al., 2022; Han et al., 2021). According to the analysis of phosphorus-dissolving and siderophore production carrier activity, it was found that BJ1 lacks both iron-producing carrier and organic phosphorus-dissolving capacity, but possesses the ability to dissolve inorganic phosphorus. This suggests that BJ1 strain is capable of converting inorganic phosphorus into soluble forms to support *B. striata* seeds absorption and utilization of phosphorus, thereby promoting their germination and growth (Long et al., 2016).

Mycorrhizal fungi are known to release symbiotic signaling factors, including plant hormones and related compounds, which activate genes associated with plant symbiotic pathways. This process ultimately facilitates the colonization of cortical cells in orchid tissue by mycorrhizal fungi, thereby promoting seed germination and growth (Favre-Godal et al., 2020). IAA is a crucial phytohormone responsible for various aspects of plant growth and development, including cell growth, root initiation, tropism, fruit ripening, and senescence. Numerous studies have shown that mycorrhizal fungi possess the capability to produce IAA and other plant growth hormones that enhance seed germination and overall plant development (Wang et al., 2021). In addition to IAA production, mycorrhizal fungi can produce gibberellins (GAs) and cytokinin. For instance, *Fusarium* sp., has been shown to generate GAs (Wu et al., 2002). To assess whether BJ1 has the ability to produce IAA, a Salkowski test was performed. The results indicated a clear positive reaction in the fermentation supernatant containing L-tryptophan; however, there was almost no positive reaction observed in the fermentation supernatant without L-tryptophan supplementation. This suggests that the BJ1 strain possesses genes related to IAA synthesis and can utilize L-tryptophan as a substrate for its production. HPLC analysis further confirmed BJ1’s capability to produce IAA. However, when L-tryptophan was not provided as a substrate, the IAA production by BJ1 was significantly reduced both inside and outside the mycelia compared to the culture condition where L-tryptophan was present (**Figure 6**). However, the ability of BJ1 strain to secrete IAA (0.0217 mg/mL) was significantly higher than that of other endophytic bacteria (0.0069 mg/mL) (Wu et al., 2022). It is known that L-tryptophan acts as a precursor for IAA biosynthesis through two distinct pathways: one dependent on L-tryptophan and another independent pathway (Wu et al., 2002; Idris et al., 2007). Evidently, BJ1 can promote IAA synthesis when utilizing L-tryptophan as a substrate, in line with existing literature (Pham et al., 2019). However, it remains uncertain whether BJ1 can independently produce IAA without relying on the L-tryptophan pathway due to potential residual presence of L-tryptophan in PDA medium. Furthermore, based on HPLC analysis and reference to standard plant growth hormone compounds, no other auxins were detected. This suggests at least partially, BJ1 promotes the germination and growth of *B. striata* seeds by secreting IAA.

We also evaluated the growth-promoting effects of BJ1. Notably, BJ1 exhibited a remarkable enhancement in growth, especially during the fourth week (**Figures 7**, **8**), which aligns with findings related to mycorrhizal fungi within the same genus (Gao et al., 2019; Li et al., 2017). Given that L-tryptophan serves as a precursor for IAA synthesis in both plants and fungi, we supplemented the medium with L-tryptophan and monitored the growth of *B. striata* seeds. As expected, the addition of L-tryptophan resulted in enhanced seed growth compared to the control group (**Figure 10**); although this effect was less pronounced than that observed with BJ1. This could be attributed to BJ1’s capacity not only to synthesize IAA but also provide nutrients conducive to seed growth; it may also stem from seeds being less efficient than BJ1 in converting L-tryptophan into IAA. Moreover, co-culturing BJ1 with L-tryptophan led to as significant increase in both the growth and development of *B. striata* seeds. Particularly noteworthy was the remarkable synergistic effect on seed length and weight, indicating potential benefits for enhancing *B. striata* seedling cultivation. These mechanisms, especially how mycorrhizal fungi synthesize IAA, are important to understand the communication between these fungi and their host (Mehmood et al., 2019, 2020). However, the current study did not explore the regulation of genes associated with IAA biosynthesis in BJ1 and *B. striata* seeds, which offers new directions for future research. Furthermore, no significant difference in seedling growth was observed between the non-symbiotic group during the third and fourth weeks. In contrast, the symbiotic group with BJ1 continued to demonstrate pronounced growth effects (**Figure 8**). This phenomenon may be attributed to the promotion of plant hormone synthesis, such as IAA, by the BJ1 symbiosis or to the nutritional benefits provided by BJ1. Additional well-designed experiments are necessary to elucidate the underlying mechanisms. In addition, the germination of Orchidaceae seeds in association with *Tulasnella* sp. fungi was also influenced by environmental factors, including pH and temperature. Noushka and Lawrie (2018) identified two distinct *Tulasnella* fungal lineages that are specific to different habitats. One of these lineages was found to be more favorable for seed germination at lower temperatures (12°C-16°C) during the fall and winter seasons. They also found that adult orchid growth was inhibited when co-planted with certain common symbiotic orchid species. In this study, controlled laboratory conditions—specifically temperature, humidity, and pH—were employed to investigate the effects of the BJ1 strain on seed germination. The temperature suitable (25°C-27°C) for germination and growth of *B. striata* seeds was mainly considered. Unfortunately, the impact of the BJ1 strain on seed germination was not evaluated under natural conditions. Future field tests will be conducted to validate these findings. Numerous studies have demonstrated that mycorrhizal fungi exert both direct and indirect influences on the growth, development, and accumulation of secondary metabolites in plants through a variety of biological activities. Direct mechanisms involve the production of auxins (such as IAA, cytokinins, gibberellins) as well as carriers for phosphorus solubilization and iron production by mycorrhizal fungi. Indirect mechanisms encompass inhibiting factors that interfere with plant growth and exhibiting antibacterial activity. These biological activities enable mycorrhizal fungi to establish beneficial relationships with plants (Long et al., 2016; Li et al., 2017; Yan et al., 2019; Wu et al., 2022). Investigations into the diversity of mycorrhizal fungi within Orchidaceae have revealed that *Tulasnella* species are dominant in promoting plant growth (Gao et al., 2019; Li et al., 2017). Some reports suggest that this genus may influence plant secondary metabolism (Ghirardo et al., 2020) and also plays a role in nitrogen transfer and transport during plant development (Fochi et al., 2017). In addition, it has been reported that *Tulasnella* sp. fungi play a crucial role in enhancing nutrient uptake and stress tolerance in plants. This is particularly evident through their symbiotic relationships, which improve root function, facilitate nutrient absorption, and bolster resilience to various environmental challenges (Jian et al., 2022). Whether BJ1 exhibits these effects warrants further exploration. Additionally, the enhanced ability of strain BJ1 to produce IAA through L-tryptophan supplementation may contribute significantly to future advancements in biological fertilizer development.

## Conclusion

In general, the isolated mycorrhizal fungus BJ1 from *B. striata*, is classified as a new species within the genus *Tulasnella* sp., significantly increased the germination rate of *B. striata* seeds from 93.04% to 96.07% by the fourth week of symbiosis. Furthermore, it enhanced the proportion of protocorms that progressed to stage 5 from 50.43% to 74.23%. Additionally, the length, width, and fresh weight of protocorms in the symbiotic group were 2.2 times, 1.8 times, and 3.7 times greater than those in the non-symbiotic group, respectively. However, there exists a limited systematic understanding of the symbiotic germination mechanisms employed by such mycorrhizal fungi. The current work demonstrates that BJ1 promotes the growth of *B. striata* seeds at least partially through the dissolution of inorganic phosphorus and the production of plant auxin IAA. In addition, when combined with L-tryptophan, BJ1 exhibits a synergistic effect on promoting both growth and development in *B. striata* seeds, which holds significant implications for the efficient cultivation of *B. striata* seedlings. Nevertheless, further elucidation is required to clarify the underlying mechanisms governing symbiotic germination between BJ1 and *B. striata* seeds. Additionally, it remains necessary to explore whether BJ1 can effectively promote seed germination and growth, enhance resistance against pathogens in *B. striata* and its potential impact on the quality of traditional Chinese medicine derived from *B. striata* in soil-based trials.

## Author’s note

The research and collection of materials for the seed plant of *B. striata* mentioned in this article comply with relevant institutional guidelines and legislation in China. All methods are carried out in accordance with the relevant guidelines in the methods section. Professor Shuili Zhang from Zhejiang University of Traditional Chinese Medicine officially identified the plant materials used in the study. Our team has obtained permission to collect plant materials. The voucher number (20221021-ND) and seeds are stored in the laboratory of the Institute of Traditional Chinese Medicine Resources, Zhejiang University of Traditional Chinese Medicine. The storage temperature of seeds is 4°C.

## Data Availability

The original contributions presented in the study are included in the article/**Supplementary Material**. Further inquiries can be directed to the corresponding authors.
