# Supplementary material for: Mycorrhizal fungus BJ1, a new species of Tulasnella sp.: its biological characteristics and promoting effect on seed germination of Bletilla striata
Source: Front Plant Sci. 2025 Feb 21;16:1542585. doi: 10.3389/fpls.2025.1542585 (PMC11885232; doi:10.3389/fpls.2025.1542585)
Supplement: Supplementary file 1 [file DataSheet1.pdf]

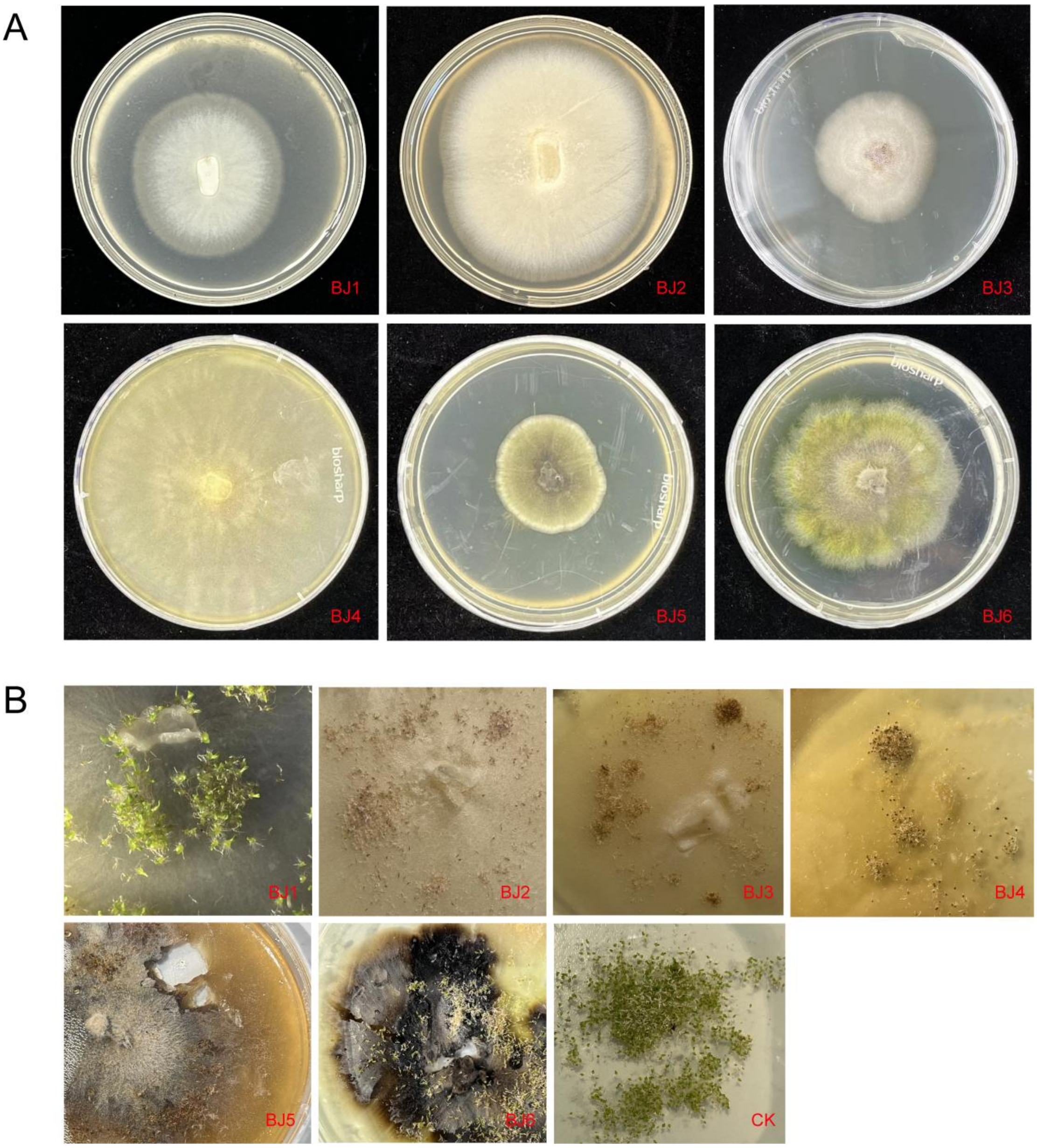

Figure S1: Isolation and screening of mycorrhizal fungi promoting germination and growth of *B. striata* seeds from *B. striata* Roots. (A) The colony morphology of 6 strains of mycorrhizal fungi isolated and purified from the roots of *B. striata*. Briefly, the fresh roots of *B. striata* were rinsed with running water, then sequentially sterilized with 70 % ethanol (10 s), 0.1% $HgCl_2$ (4 min) and finally washed with sterile water. The sterilized roots were cut into 2-3 mm thin slices, place on the PDA medium, and cultured at 27 °C. Mycorrhizal fungi were isolated and purified by the top purification method of mycelium, and a total of 6 mycorrhizal fungi were obtained and temporarily named BJ1-BJ6. (B) Test on the ability of mycorrhizal fungi to promote the growth and germination of *B. striata* seeds. The purified strains were inoculated into PDA solid medium, and then sprinkled with sterile seeds respectively, and cultured under 12 h light and 12 h dark conditions for 2 weeks, the seed growth and germination promotion effects of fungi were observed, and those seeds without fungi co-culture were as control (CK). Among the 6 strains of fungi isolated, only BJ1 showed a significant role in promoting the germination and growth of *B. striata* seeds.

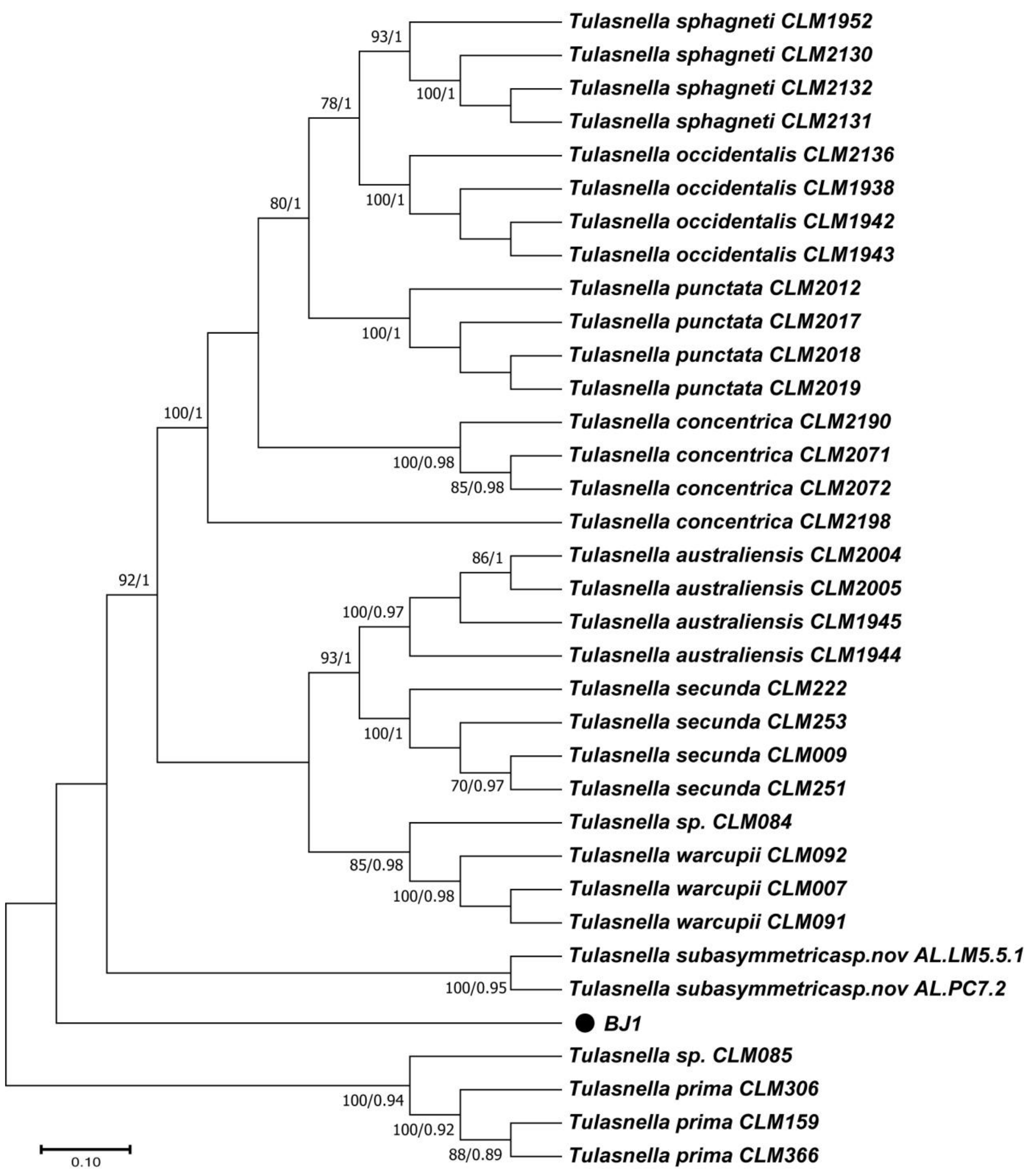

Figure S2: BI tree for *Tulasnella* using the C3304 region. Numbers on the branches are bootstrap (>70%)/BPP (>0.70) support values.

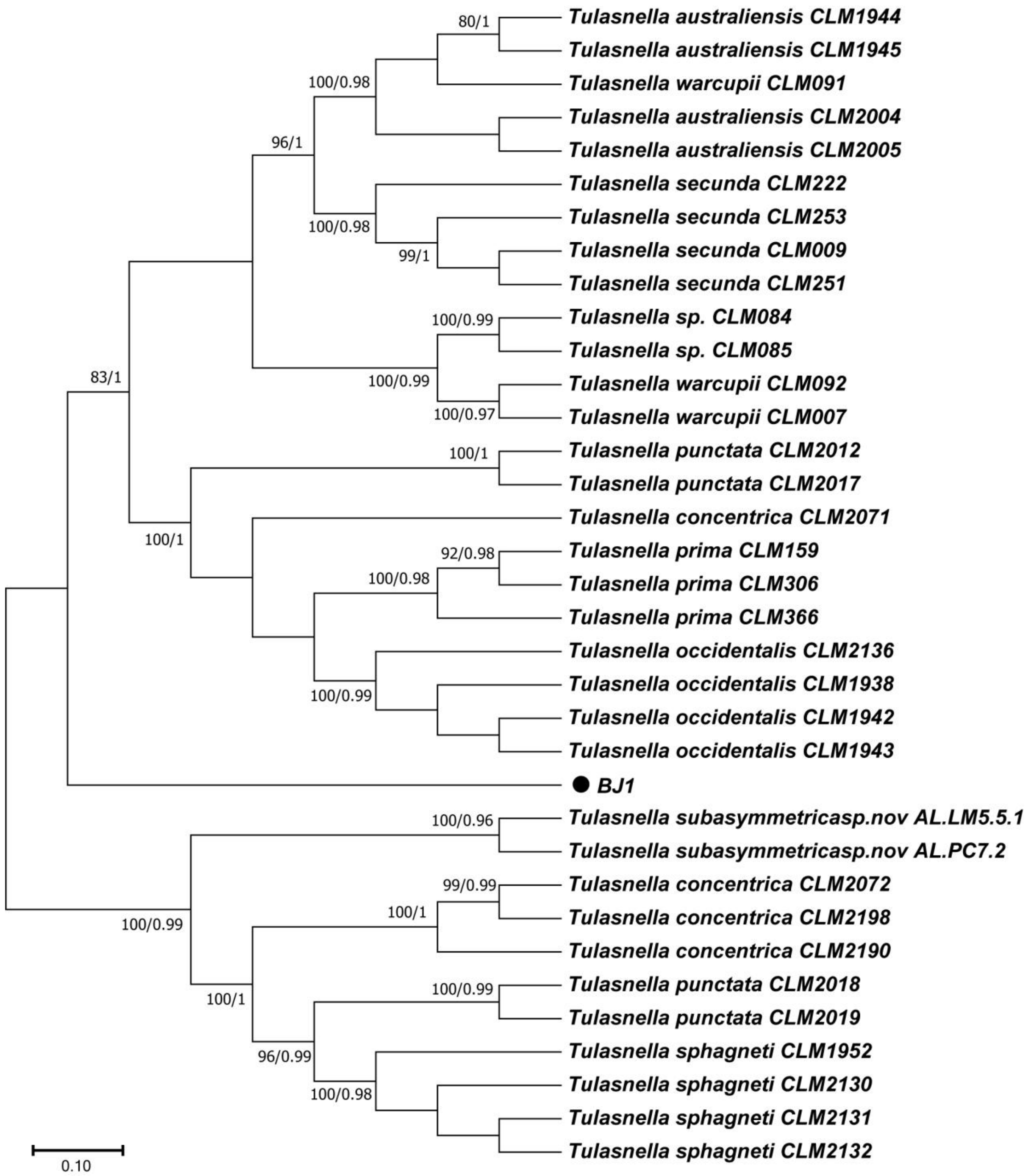

Figure S3: BI tree for *Tulasnella* using the C4102 region. Numbers on the branches are bootstrap (>70%)/BPP (>0.70) support values.

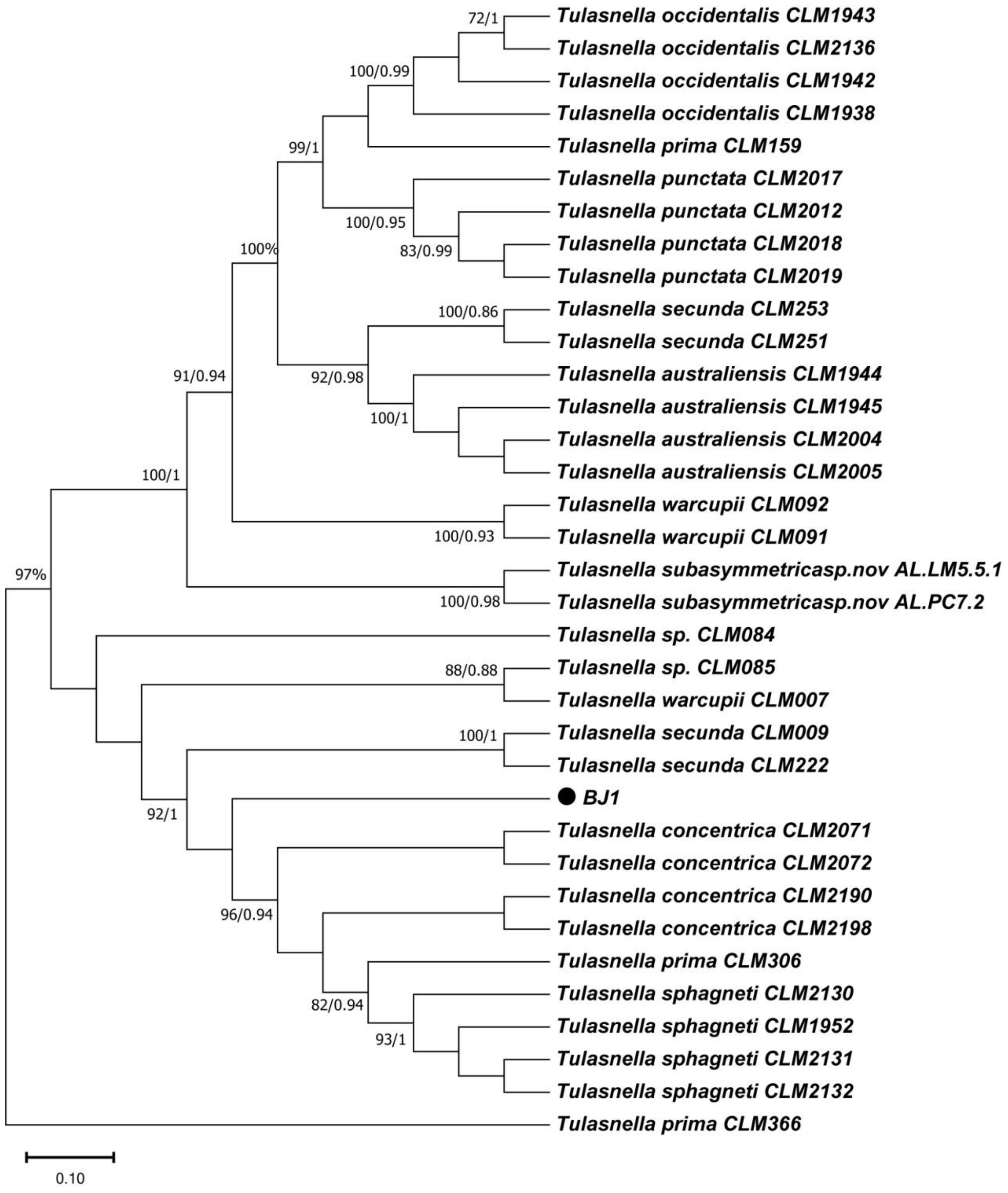

Figure S4: BI tree for *Tulasnella* using the C14436 region. Numbers on the branches are bootstrap (>70%)/BPP (>0.70) support values.

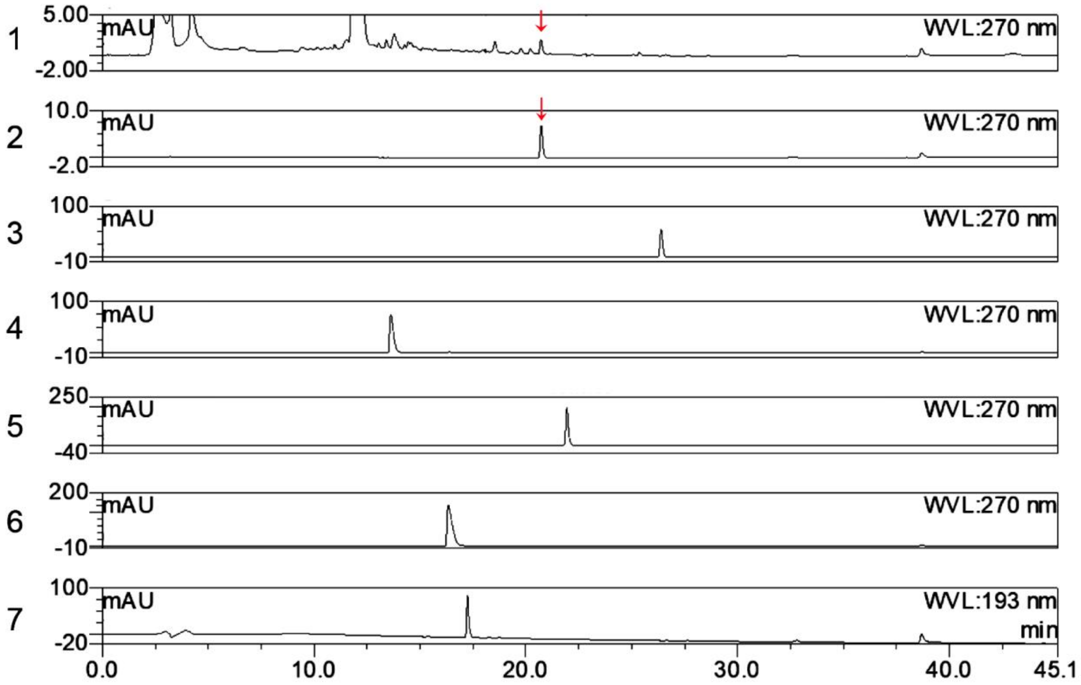

Figure S5: Detection of plant hormones in BJ1 fermentation broth. 1 shows the concentrated extract from the BJ1 fermentation broth supplemented with L-tryptophan, while 2-7 represent standards for indole-3-acetic acid (IAA), naphthaleneacetic acid (NAA), kinetin (KT), abscisic acid (ABA), 6-benzylaminopurine (6-BA), and gibberellic acid (GA), respectively.
